# Supplementary figures and images for: Captivity causes taxonomic and functional convergence of gut microbial communities in bats
Source: PeerJ. 2019 Apr 30;7:e6844. doi: 10.7717/peerj.6844 (PMC6499062; doi:10.7717/peerj.6844)

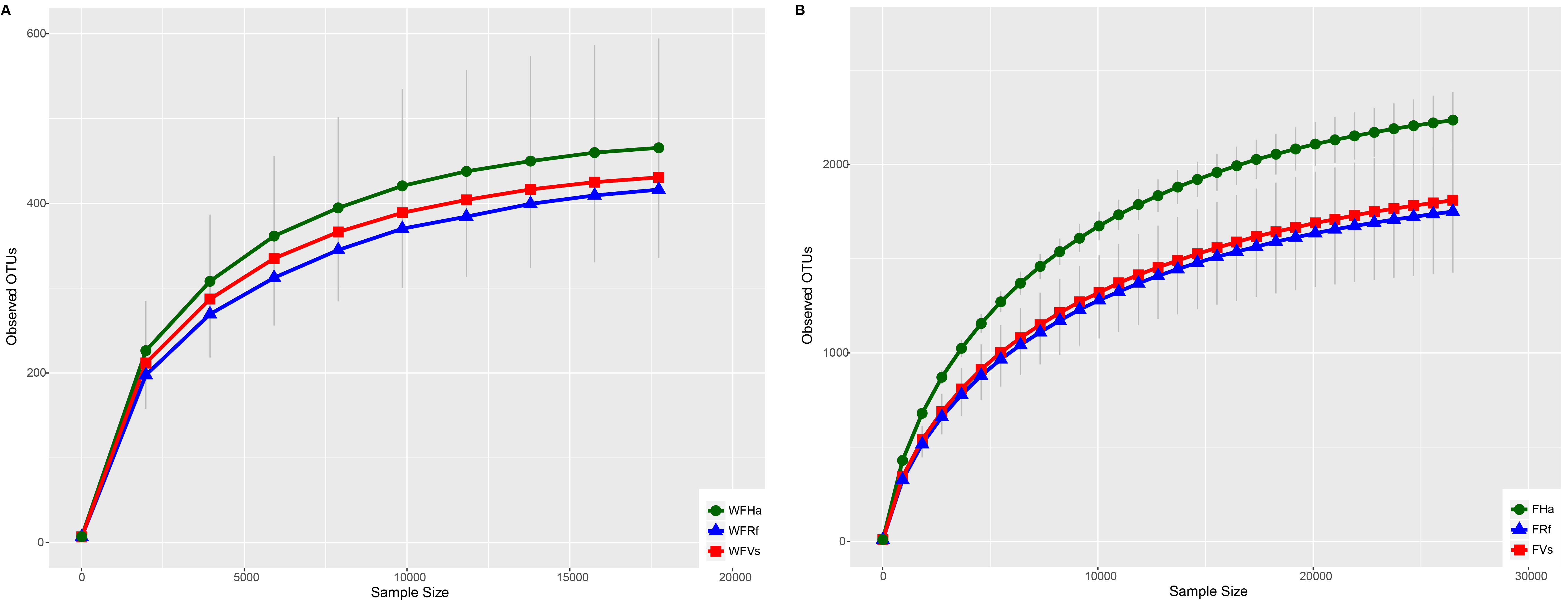

Supplement: Figure S1 — (A) Wild bats (B) Captive bats. Points are means ± SE, with the numbers of bats per group shown in Table 1. The meanings of WFVs, WFRf, WFHa, FVs, FRf and FHa are same as in Fig. 1, (see Fig. 1 legend). [file peerj-07-6844-s001.png]

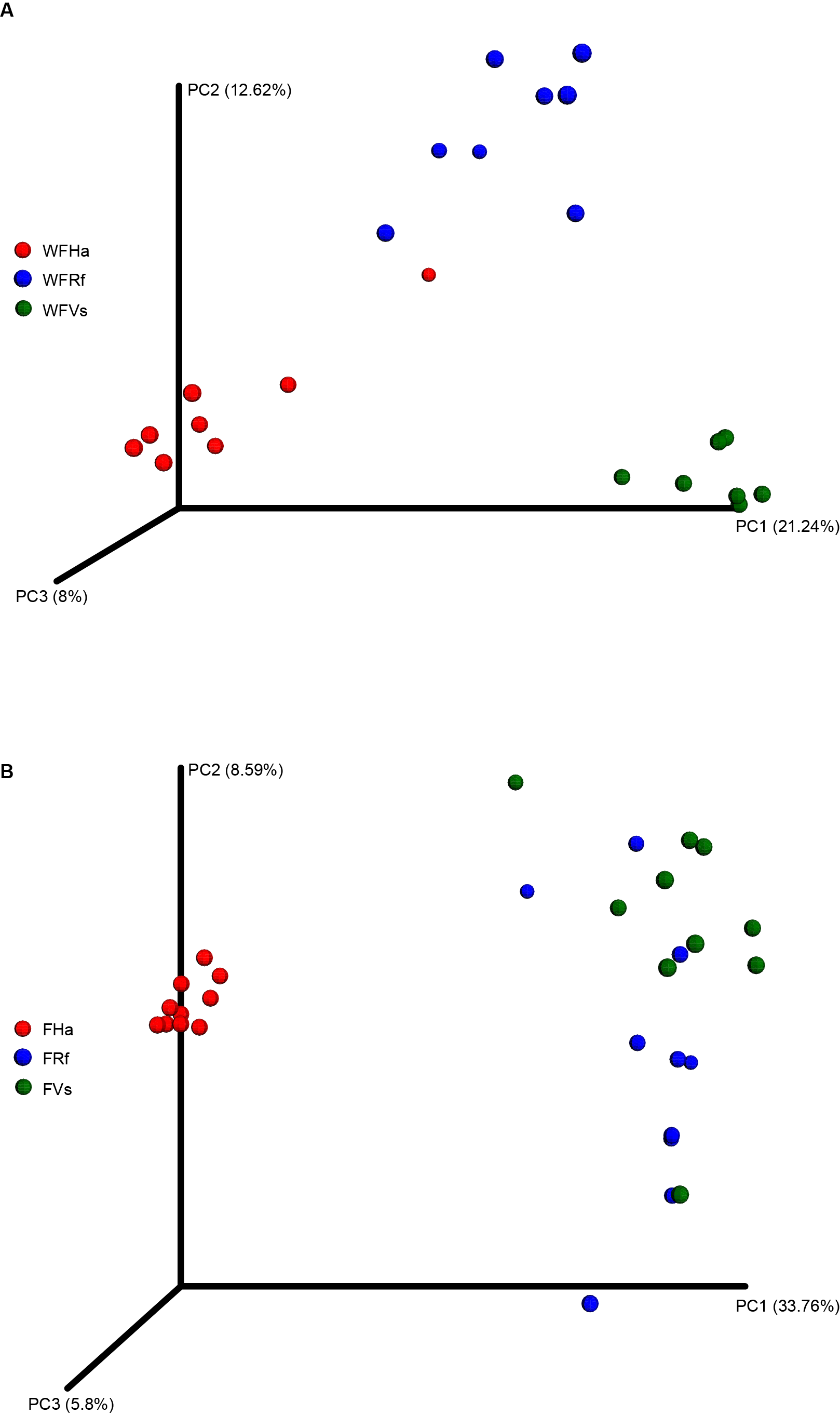

Supplement: Figure S2 — Wild (A) and captive (B) bats’ fecal bacterial communities clustered using principal coordinates analysis. Each point corresponds to a fecal sample colored according to bat species with different symbols corresponding to host family (red circle, Hipposideridae, green square, Vespertilionidae, blue triangle, Rhinolophidae). [file peerj-07-6844-s002.png]

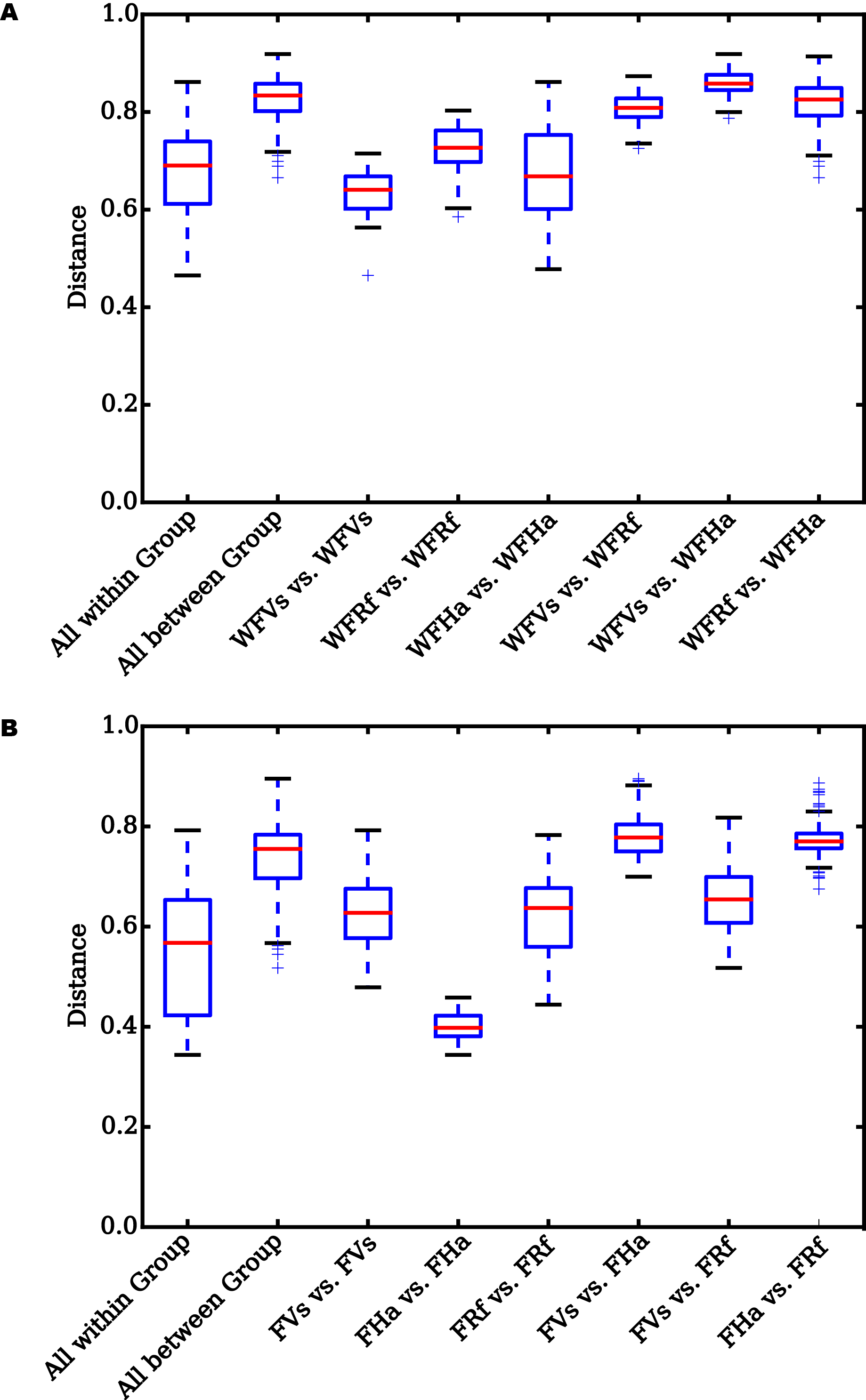

Supplement: Figure S3 — X-axis, pairwise comparisons among groups. Y-axis, UniFrac distances. Box borders represent upper and lower interquartile ranges. Red lines, whiskers, and “+” represent the median values, 1.5 times the interquartile range beyond upper and lower quartiles, and outliers respectively. Significant differences in the UniFrac distances for pairwise comparisons among groups are shown in Table 2. WFVs, WFRf, WFHa, FVs, FRf and FHa are defined in the legend for Fig. 1. If the distance between two groups is significantly greater than that within the groups, the difference between these groups is significant. [file peerj-07-6844-s003.png]

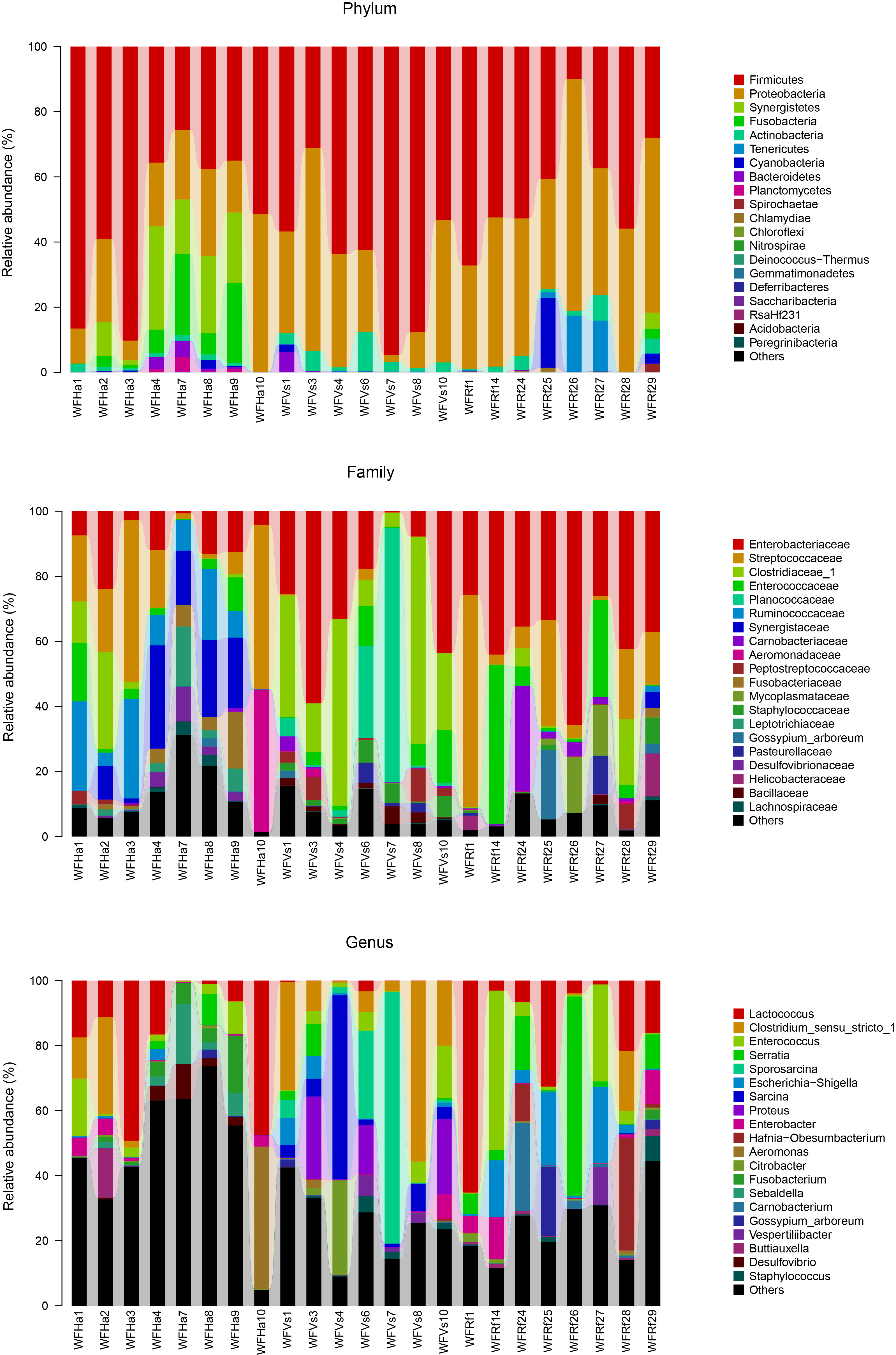

Supplement: Figure S4 — WFVs, WFRf and WFHa represent fecal samples from V. sinensis, R. ferrumequinum and H. armiger collected from the wild respectively. [file peerj-07-6844-s004.png]

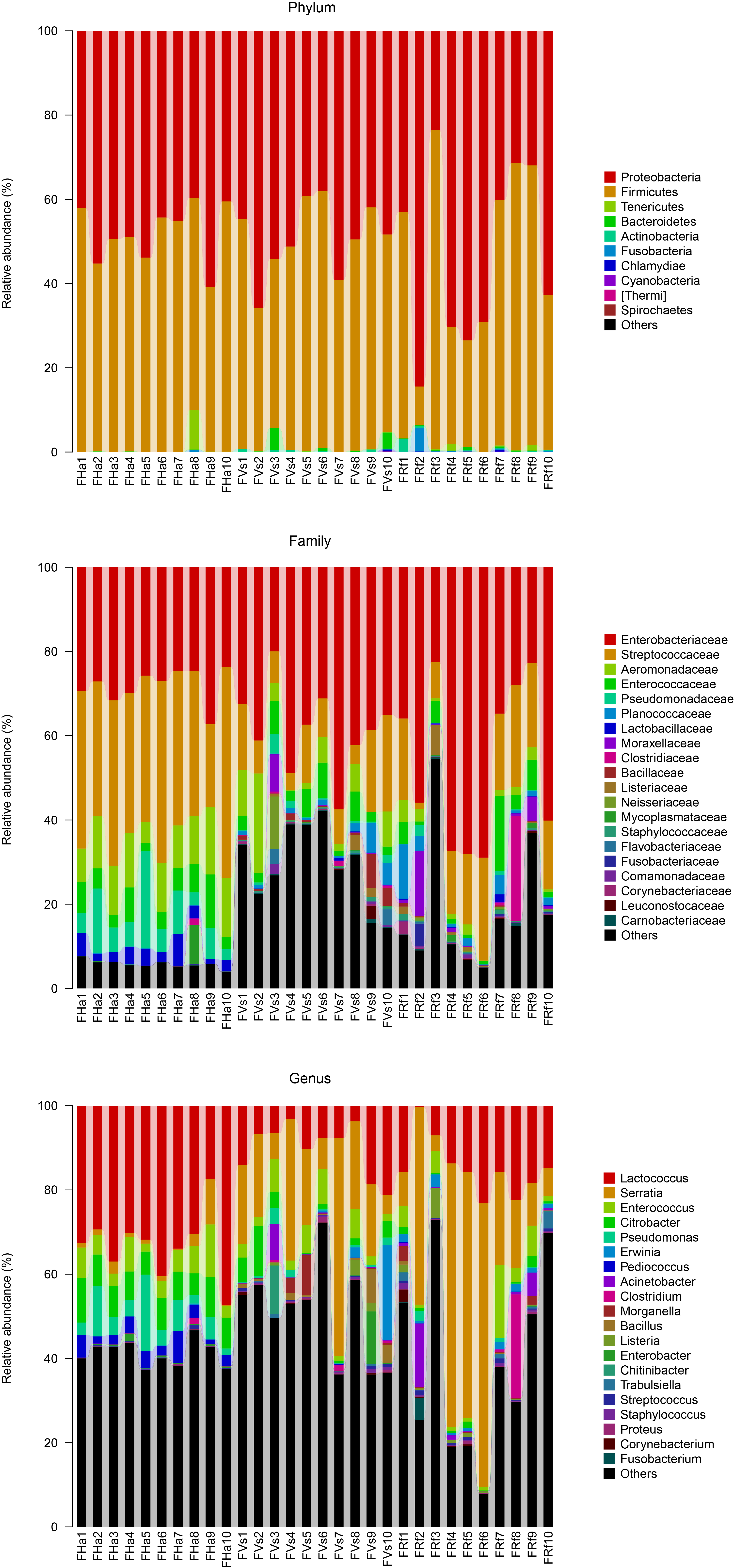

Supplement: Figure S4 — FVs, FRf and FHa represent fecal samples from captive V. sinensis, R. ferrumequinum and H. armiger respectively. [file peerj-07-6844-s005.png]
